# Bone Scintigraphy versus PSMA-Targeted PET/CT or PET/MRI in Prostate Cancer: Lessons Learned from Recent Systematic Reviews and Meta-Analyses

**DOI:** 10.3390/cancers14184470

**Published:** 2022-09-15

**Authors:** Francesco Dondi, Domenico Albano, Francesco Bertagna, Giorgio Treglia

**Affiliations:** 1 Division of Nuclear Medicine, University of Brescia and ASST Spedali Civili Brescia, 25123 Brescia, Italy; 2 Clinic of Nuclear Medicine, Imaging Institute of Southern Switzerland, Ente Ospedaliero Cantonale, 6500 Bellinzona, Switzerland; 3 Department of Nuclear Medicine and Molecular Imaging, Lausanne University Hospital and University of Lausanne, 1015 Lausanne, Switzerland; 4 Faculty of Biomedical Sciences, Università della Svizzera italiana, 6900 Lugano, Switzerland

Positron emission tomography (PET) combined with computed tomography (PET/CT) or magnetic resonance imaging (PET/MRI) using several radiopharmaceuticals—particularly prostate-specific membrane antigen (PSMA)-targeting radiopharmaceuticals—are new-generation imaging modalities for prostate cancer (PC) staging and restaging. These whole-body imaging methods combining functional and anatomical information increase diagnostic accuracy in detecting PC lesions compared to conventional imaging [1].

Regarding bone scintigraphy with ^99m^Tc-diphosphonates, this imaging method may detect lesions missed by CT in some cases; however, its specificity is not adequate as benign lesions causing increased osteoblastic activity can be mistaken for PC metastases. In detecting bone metastases at the initial diagnosis of PC, bone scintigraphy presents a relatively low diagnostic yield (3.5% with serum PSA values ≤10 ng/mL, 6.9% with serum PSA between 10 and 20 ng/mL, and 41.8% with serum PSA >20 ng/mL) [2]. Furthermore, bone scintigraphy with ^99m^Tc-diphosphonates only examines the bones and does not provide information on extraosseous (i.e., lymphatic or visceral) metastases in PC [2].

PSMA is a membrane antigen that is overexpressed in the majority of PC cells and it is an ideal target for PC diagnosis and therapy (theranostics). In particular, PSMA-targeted PET/CT or PET/MRI (using PSMA-targeting radiopharmaceuticals labelled with ^68^Ga, ^18^F, or ^64^Cu) is gaining importance in the staging and restaging of PC [3,4,5,6,7,8,9,10,11,12,13,14]. The accurate diagnosis of bone metastases is relevant in guiding local and systemic treatment in PC. In this regard, a network meta-analysis of 45 studies recently demonstrated that PSMA-targeting radiopharmaceuticals are the best PET tracers in terms of diagnostic accuracy in detecting bone metastases in PC [15].

As demonstrated by recent systematic reviews, several studies performed a head-to-head comparison between bone scintigraphy with ^99m^Tc-diphosphonates and PSMA-targeted PET/CT or PET/MRI in detecting bone metastases in PC [16,17,18,19]. The most recent systematic review and meta-analysis on this topic, published by Zhao and colleagues, included six studies with 546 PC patients [16]. The pooled sensitivity and specificity and 95% confidence interval values (95%CI) for bone scintigraphy were 83% (95%CI: 69–91%) and 68% (95%CI: 41–87%), respectively. The same values were significantly higher for PSMA-targeted PET/CT: 98% (95%CI: 94–99%) and 97% (95%CI: 91–99%), respectively. The diagnostic accuracy on a per-patient basis, measured as the area under the curve (AUC), was significantly higher for PSMA-targeted PET/CT (0.99; 95%CI: 0.96–1.00) compared to bone scintigraphy (0.85; 95%CI: 0.81–0.87). Interestingly, PSMA-targeted PET/CT correctly identified bone metastases in 22.3% of patients with negative bone scintigraphy, whereas bone scintigraphy correctly identified bone metastases in a limited percentage (1.9%) of patients with negative PSMA-targeted PET/CT findings. Furthermore, PSMA-targeted PET/CT provided a significant change in management compared to bone scintigraphy (e.g., through the detection of bone metastases in patients with negative bone scintigraphy, by identifying more bone metastases in oligometastatic patients using bone scintigraphy, by revealing extraosseous metastases, or by decreasing the number of false-positive findings compared to bone scintigraphy) [16]. Considering these results, the diagnostic performance of PSMA-targeted PET/CT in detecting bone metastases in PC is clearly superior to that of bone scintigraphy. Furthermore, bone scintigraphy does not offer significant additional information in patients with a previous PSMA-targeted PET/CT (even if negative); moreover, compared to PET/CT it does not provide information on extraosseous metastases in PC.

The available systematic reviews and meta-analyses on this topic show several limitations (e.g., a limited number of included studies, the retrospective study design of most included articles, heterogeneity among the included studies about PC clinical setting, PC patient characteristics, the characteristics of the index tests, and the reference standards), and some advantages (e.g., head-to-head comparisons between imaging techniques); nevertheless, it is unlikely that future studies will change the conclusions of the available evidence-based data, which suggest the limited added value of bone scintigraphy compared to PSMA-targeted PET/CT or PET/MRI in PC.

It remains unclear whether management changes based on PSMA-targeted PET compared to bone scintigraphy translate into improved patient outcomes, and whether the advantages of PSMA-targeted PET over bone scintigraphy are confirmed in each specific PC clinical setting (e.g., staging, restaging, and treatment response assessment) [20].

Beyond the advantages in the diagnostic performance of PSMA-targeted PET/CT compared to bone scintigraphy in PC, recent high-quality studies have shown the better cost-effectiveness of PSMA-targeted PET/CT compared to conventional imaging (including bone scintigraphy) [21,22].

Notably, all current treatment schemes in PC are based on conventional imaging (CT and bone scan), and it is currently not clear how and whether the metastases seen in PSMA-targeted PET, and not seen in conventional imaging, should be treated. Furthermore, data on the better outcomes for PC patients treated for the metastases seen in PSMA-targeted PET, and not in conventional imaging, are still lacking.

In conclusion, evidence-based data demonstrate that bone scintigraphy is clearly inferior to PSMA-targeted PET in terms of diagnostic accuracy for detecting PC bone metastases, and may not be needed in patients with PC who have already performed PSMA-targeted PET/CT or PET/MRI. It is likely that, in the near future, the increased availability of PSMA-targeted PET/CT or PET/MRI will strongly affect the use and the usefulness of bone scintigraphy in PC patients.

## References

[1] Annunziata S., Pizzuto D.A., Treglia G. (2020). Diagnostic Performance of PET Imaging Using Different Radiopharmaceuticals in Prostate Cancer According to Published Meta-Analyses. Cancers.

[2] Trabulsi E.J., Rumble R.B., Jadvar H., Hope T., Pomper M., Turkbey B., Rosenkrantz A.B., Verma S., Margolis D.J., Froemming A. (2020). Optimum Imaging Strategies for Advanced Prostate Cancer: ASCO Guideline. J. Clin. Oncol..

[3] Lisney A.R., Leitsmann C., Strauß A., Meller B., Bucerius J.A., Sahlmann C.O. (2022). The Role of PSMA PET/CT in the Primary Diagnosis and Follow-Up of Prostate Cancer-A Practical Clinical Review. Cancers.

[4] Zhao Y., Simpson B.S., Morka N., Freeman A., Kirkham A., Kelly D., Whitaker H.C., Emberton M., Norris J.M. (2022). Comparison of Multiparametric Magnetic Resonance Imaging with Prostate-Specific Membrane Antigen Positron-Emission Tomography Imaging in Primary Prostate Cancer Diagnosis: A Systematic Review and Meta-Analysis. Cancers.

[5] Galgano S.J., McDonald A.M., West J.T., Rais-Bahrami S. (2022). Defining Oligometastatic Disease in the New Era of PSMA-PET Imaging for Primary Staging of Prostate Cancer. Cancers.

[6] Salem A.E., Fine G.C., Covington M.F., Koppula B.R., Wiggins R.H., Hoffman J.M., Morton K.A. (2022). PET-CT in Clinical Adult Oncology-IV. Gynecologic and Genitourinary Malignancies. Cancers.

[7] Kase A.M., Tan W., Copland J.A., Cai H., Parent E.E., Madan R.A. (2022). The Continuum of Metastatic Prostate Cancer: Interpreting PSMA PET Findings in Recurrent Prostate Cancer. Cancers.

[8] Luining W.I., Cysouw M.C.F., Meijer D., Hendrikse N.H., Boellaard R., Vis A.N., Oprea-Lager D.E. (2022). Targeting PSMA Revolutionizes the Role of Nuclear Medicine in Diagnosis and Treatment of Prostate Cancer. Cancers.

[9] Neels O.C., Kopka K., Liolios C., Afshar-Oromieh A. (2021). Radiolabeled PSMA Inhibitors. Cancers.

[10] Manafi-Farid R., Ranjbar S., Jamshidi Araghi Z., Pilz J., Schweighofer-Zwink G., Pirich C., Beheshti M. (2021). Molecular Imaging in Primary Staging of Prostate Cancer Patients: Current Aspects and Future Trends. Cancers.

[11] Zhang H., Koumna S., Pouliot F., Beauregard J.M., Kolinsky M. (2021). PSMA Theranostics: Current Landscape and Future Outlook. Cancers.

[12] El Fakiri M., Geis N.M., Ayada N., Eder M., Eder A.C. (2021). PSMA-Targeting Radiopharmaceuticals for Prostate Cancer Therapy: Recent Developments and Future Perspectives. Cancers.

[13] Mokoala K., Lawal I., Lengana T., Kgatle M., Giesel F.L., Vorster M., Sathekge M. (2021). PSMA Theranostics: Science and Practice. Cancers.

[14] Hyväkkä A., Virtanen V., Kemppainen J., Grönroos T.J., Minn H., Sundvall M. (2021). More Than Meets the Eye: Scientific Rationale behind Molecular Imaging and Therapeutic Targeting of Prostate-Specific Membrane Antigen (PSMA) in Metastatic Prostate Cancer and Beyond. Cancers.

[15] Liu F., Dong J., Shen Y., Yun C., Wang R., Wang G., Tan J., Wang T., Yao Q., Wang B. (2021). Comparison of PET/CT and MRI in the Diagnosis of Bone Metastasis in Prostate Cancer Patients: A Network Analysis of Diagnostic Studies. Front. Oncol..

[16] Zhao G., Ji B. (2022). Head-To-Head Comparison of ^68^Ga-PSMA-11 PET/CT and ^99m^Tc-MDP Bone Scintigraphy for the Detection of Bone Metastases in Patients with Prostate Cancer: A Meta-Analysis. AJR Am. J. Roentgenol..

[17] Wang J., Han Y., Lin L., Zhang L., Li J., Gao H., Fu P. (2021). Systematic review & meta-analysis of positron emission tomography/computed tomography and bone scan in the diagnosis of prostate lesions. Transl. Androl. Urol..

[18] Zhao R., Li Y., Nie L., Qin K., Zhang H., Shi H. (2021). The meta-analysis of the effect of 68Ga-PSMA-PET/CT diagnosis of prostatic cancer compared with bone scan. Medicine.

[19] Zhou J., Gou Z., Wu R., Yuan Y., Yu G., Zhao Y. (2019). Comparison of PSMA-PET/CT, choline-PET/CT, NaF-PET/CT, MRI, and bone scintigraphy in the diagnosis of bone metastases in patients with prostate cancer: A systematic review and meta-analysis. Skeletal Radiol..

[20] Woo S. (2022). Editorial Comment: Time to Ditch Bone Scans for Prostate Cancer Workup in the Era of PSMA PET/CT?. AJR Am. J. Roentgenol.

[21] Song R., Jeet V., Sharma R., Hoyle M., Parkinson B. (2022). Cost-Effectiveness Analysis of Prostate-Specific Membrane Antigen (PSMA) Positron Emission Tomography/Computed Tomography (PET/CT) for the Primary Staging of Prostate Cancer in Australia. Pharmacoeconomics.

[22] de Feria Cardet R.E., Hofman M.S., Segard T., Yim J., Williams S., Francis R.J., Frydenberg M., Lawrentschuk N., Murphy D.G., De Abreu Lourenco R. (2021). Is Prostate-specific Membrane Antigen Positron Emission Tomography/Computed Tomography Imaging Cost-effective in Prostate Cancer: An Analysis Informed by the proPSMA Trial. Eur. Urol..

